# ‘Our training didn't prepare us for private practice’: A multi‐method study of dietetics graduates' preparedness for private practice employment

**DOI:** 10.1111/1747-0080.70020

**Published:** 2025-05-20

**Authors:** Merran Blair, Charlotte E. Rees, Simone Gibson, Lana J. Mitchell, Ella Ottrey, Lynn V. Monrouxe, Claire Palermo

**Affiliations:** ^1^ Department of Nutrition, Dietetics and Food Monash University Notting Hill Victoria Australia; ^2^ Faculty of Medicine, Health and Life Science Swansea University, Park Campus Swansea UK; ^3^ Monash Centre for Scholarship in Health Education, Faculty of Medicine, Nursing and Health Sciences Monash University Clayton Victoria Australia; ^4^ School of Clinical Sciences Monash University Clayton Victoria Australia; ^5^ School of Allied Health Sciences Griffith University Gold Coast Queensland Australia; ^6^ Faculty of Medicine and Health The University of Sydney Sydney New South Wales Australia; ^7^ Faculty of Medicine, Nursing and Health Sciences Monash University Clayton Victoria Australia

**Keywords:** dietitian, employability, graduate, landscapes of practice, private practice

## Abstract

**Aim:**

This multi‐method study explored dietetics graduates' preparedness for the landscape of private practice employment.

**Methods:**

Qualitative, in‐depth interview and audio‐diary data were collected longitudinally in 2019 regarding dietetics graduates' experiences of private practice employability. Framework analysis of qualitative data prompted a quantitative survey of university representatives in 2021–2022 on the use of private practice placements. Survey data were analysed descriptively. Qualitative themes were reviewed alongside quantitative findings and were interpreted in the context of the sociocultural theory, *landscapes of practice*.

**Results:**

Qualitative data from nine dietetics graduates (total 12 hours of audio data) indicated unpreparedness for this setting, with the following themes identified: 1) private practice skills were lacking; 2) making a living from private practice was challenging; and 3) support was needed. Quantitative data from 18 program directors of accredited universities (100% response) illustrated that private practice placement experiences varied from <10 to 40 days. Placements were most commonly elective (44%) and were not offered by four programs (22%). University program directors expressed concerns that private practice placements were challenging to organise and offered limited client contact hours.

**Conclusions:**

It is an educational priority to prepare graduates for available employment opportunities by providing learning experiences that traverse the dietetics landscape of practice. Co‐designing placements with private practice business owners may support authentic experiences of appropriate durations, with ample opportunities for students to build skills to enhance preparedness for this growing employment setting.

## 1 INTRODUCTION

Private practice is a growing employment setting for Australian dietitians,^1^, ^2^ and is the most common employment setting for new graduates.^3^, ^4^ Graduates working in private practice, however, feel underprepared and need additional education and support.^3^, ^5^, ^6^ Despite this, there is limited literature about the experiences of newly qualified dietitians working in private practice. No published research addresses graduate preparedness for private practice as the primary study aim, with only minor details reported as a subset of larger results.^3^, ^4^, ^5^, ^7^, ^8^, ^9^, ^10^ Private practice has different demands compared to public settings, such as managing business operations, client acquisition, and billing systems.^10^, ^11^ Ensuring graduates have the skills, knowledge, and confidence to navigate this setting is vital for maintaining positive client outcomes. Furthermore, graduates who are ill‐prepared for private practice may experience job dissatisfaction or desires to leave the profession, impacting workforce sustainability.^12^, ^13^, ^14^ This incongruence between education and graduate employment is concerning yet has not been adequately explored to date.^9^

The limited research examining employment in dietetics private practice has shown benefits, such as flexibility and independence of work, which become prominent once businesses are established.^8^ However, a range of challenges exists, including concerns about managing time and money, as well as experiencing a lack of support, isolation, and competition with peers.^8^, ^15^ New graduates experience a steep learning curve when starting out in private practice.^7^ They are more likely to work longer hours, charge less than established dietitians, and experience pressure from referrers to charge less than adequate fees.^7^ Experienced dietitians perceive that graduates charging suboptimal fees reflect poorly on the profession.^8^ To understand these interrelated issues, there is a need to delve deeper into graduate dietitians' experiences of private practice.

Practice‐based learning, or work placement, forms a key component of dietetics education. In Australia and New Zealand, a minimum of 100 days of work placement is required.^16^, ^17^ While placements have traditionally occurred in the public sector, there has been a call for more diverse placement settings, including private practice.^6^, ^18^ In support of greater diversity, the Australian Accreditation Standards for dietetics education programs were changed in 2022, stating that within the 100 days of placement, universities must offer ‘a variety of workplace learning experiences reflecting socio‐ecological approaches to health, major health priorities and the broad landscape of dietetics practice’.^19^ Securing placements for increasing numbers of health professional students can be challenging,^20^ and incorporating diverse placement locations may help attenuate this. Some universities are known to provide private practice placements within their degrees, but it is unclear how many. This contextual information is needed to understand graduates' preparedness for private practice and the implications for the dietetics workforce.

Wenger‐Trayner et al.'s *landscapes of practice* describes the settings in which health professionals work as a series of interconnected communities, each with its own history and regime of competence.^19^ The culmination of different communities creates a landscape of practice.^19^ Individuals are generally members of multiple landscapes and engage in what is described as multi‐membership.^19^ Professional proficiency requires individuals to be skilled at navigating the different communities within the landscape and the boundaries between communities, or boundary crossing.^19^ Students must navigate multiple landscapes as they bridge the divide between academia and the settings in which they will learn and work.^19^, ^21^ Private practice represents both a community within the broader landscape of dietetic practice and a landscape in and of itself.

Health professions education is competency‐focused.^22^ However, successfully navigating a landscape of practice goes beyond professional competence and requires the development of ‘knowledgeability’, which involves translating competencies into a ‘meaningful moment of service’ across settings within the landscape.^19^ For example, while learners may develop competence in managing an individual's nutrition care in hospital, this may not translate to private practice. Identification, or ‘becoming a person who inhabits the landscape’,^19^ is essential to creating knowledgeability. Identification can be achieved through a combination of engagement (participation within the landscape), imagination (conceptualising the landscape and how the individual fits within it) and alignment (fitting in with, and contributing to, the accepted activities within the landscape).^19^ Students who engage with activities within the landscape and navigate between communities across the landscape more readily establish knowledgeability, likely leading to enhanced preparedness for employment.^19^ The landscapes of practice theory suggests that private practice placement experiences would better prepare dietetics graduates to work in this setting.^19^

We aimed to explore new graduates' experiences of employment and employability within the landscape of private practice. We addressed two research questions: (1) What are dietetics graduates' experiences of preparation for employment in the landscape of private practice? (2) Can current education program placements partly explain these experiences?

## 2 METHODS

**FIGURE 1:**
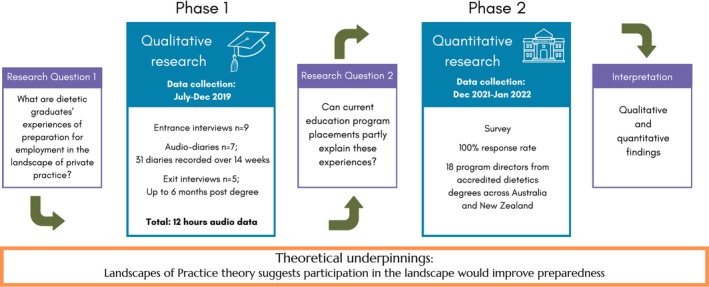
The multi‐method design and the research questions guiding each data collection stage regarding private practice preparedness for dietetics graduates, underpinned by the landscapes of practice theory.

We utilised a multi‐method study design, employing qualitative methods to answer the first research question and quantitative methods to answer the second (see Figure 1).^23^ The qualitative phase (completed as part of a larger program of work^5^, ^24^, ^25^, ^26^) identified issues warranting further investigation, informing the quantitative phase (Figure 1). Our research was underpinned by interpretivism^27^; we sought subjective understanding of the multiple realities of graduates and universities in the context of private practice preparedness.^28^ Ethics was granted by Monash University's Human Research Ethics Committee (Project IDs 20 026 and 24 556). In undertaking and reporting our study, we employed the Good Reporting of A Mixed Methods Study (GRAMMS) checklist.^29^

Our research team included diverse philosophical standpoints (including post‐positivist, interpretivist and pragmatist perspectives), which supported reflexivity during data analysis.^30^ Our first author was undertaking a PhD in graduate employability and maintained a reflexive journal, which was regularly discussed with the supervisory team (SG, LJM, CP) to assess assumptions and perspectives influencing the study. Our research team was experienced in health professions education and research and included five dietitians. Our first author had worked in private practice as a new graduate and, during this research, worked as a clinical educator in a private practice teaching clinic.

Recruitment for the qualitative phase was purposive and occurred via email, virtual posts, face‐to‐face, and snowballing, whereby participants were encouraged to nominate peers to invite. Our sample for this paper included nine dietetics graduates from a single Australian university (taken from a larger sample of medicine (*n* = 12), nursing (*n* = 7), pharmacy (*n* = 7) and dietetics (*n* = 9) graduates).^24^, ^25^, ^26^ We conducted entrance interviews at the end of participants' degrees (prior to them entering the workforce), longitudinal audio‐diaries during the early new graduate phase, and exit interviews at the study conclusion, approximately 6 months post‐degree completion; all audio‐recorded. Our interview questions and weekly audio‐diary prompts asked what ‘preparedness for practice’ meant to participants and for them to recount times when they felt prepared or unprepared for practice. We only analysed dietary data as these were the only participants to discuss employability issues. Our analysis produced substantial results, including broad experiences of employability.^5^ The more specific topic of employability in private practice warranted in‐depth discussion, which we address in this paper.

We transcribed audio data verbatim and uploaded transcripts into NVivo software (V13 (2020, R1), QSR International). We analysed data using a framework method,^31^ whereby one author coded data from one entrance interview, one set of audio‐diaries, and one exit interview. Three other authors concurrently coded one entrance interview, one set of audio‐diaries, or one exit interview, before comparing coding and creating a coding framework. We discussed the resultant framework as a team, and additional analytical insights supported further development of the coding framework. We then condensed codes into themes and sub‐themes,^32^ and explored differences over time.^33^ We summarised participants' experiences of employment, or offers of employment, in private practice longitudinally in pen portraits.^33^ We have replaced participant names with pseudonyms.

Our qualitative results informed the quantitative phase. We developed an online survey in Qualtrics XM (Provo, UT) to better understand the use of private practice placements in Australia and New Zealand dietetics education (survey available upon request). We distributed the survey to program directors of all 18 Australia and New Zealand universities offering accredited dietetics degrees at the time of the study^34^, ^35^ via email, with two reminders. We offered categorical responses for university, whether placements or internship experiences were offered (now or in the future), if it was a core or elective part of the curriculum, and if the experience was provided by an external company. We employed free text responses for how the placement would be changed in the future, the number of students, and how many days the students attended. In addition, we invited participants to share free text responses about anything else related to private practice placements.

Using SPSS software (IBM Corp. Released 2021. IBM SPSS Statistics for Windows, Version 28.0. Armonk, NY: IBM Corp.), we analysed categorical data using descriptive statistics. One author summarised the 12 free text responses, which were reviewed by our team.

During the interpretative phase, we utilised the survey results to further explain the qualitative findings by comparing and contrasting themes from the qualitative analysis with key findings from the survey. For example, the qualitative themes that described preparedness for private practice were further interpreted and verified from the survey data describing the number of universities offering a private practice placement and the nature of that experience. We then interpreted all findings with respect to the landscapes of practice theory.^19^ We considered theory elements such as identification and knowledgeability,^19^ and whether they were present in the graduates' reported experiences and the potential to build these based on the experiences offered by universities.

## 3 RESULTS

Nine dietetics graduates participated in the qualitative phase. Participants had a median age of 23 years (range 22–27 years), were mostly female (*n* = 8; 89%), spoke English as their first language (*n* = 7; 78%) and identified as Oceanian (*n* = 5; 56%).^36^ Graduates reported feeling unprepared for private practice, and longitudinal analysis identified no differences in participants' perspectives over time. We provide four pen portraits in Table 1 to demonstrate different experiences of private practice employment.

**TABLE 1:** Pen portraits of four participants' experiences of private practice employment as dietetics graduates up to 6 months post‐degree completion.

Participant^a^	Summary of their experiences with private practice over the course of the study
Caitlin 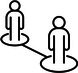	Caitlin applied for, and was offered, a role as the sole contractor dietitian for a National Disability Insurance Scheme provider. Concerned about the lack of support with no other dietitians on staff and the likelihood of managing highly complex clients, they turned the position down.
Pat 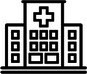	Pat worked for several months in a large dietetic private practice business owned by a dietitian. They felt unsupported and found it challenging to find enough clients to generate an adequate income. They used the experience gained to bolster subsequent job applications and transitioned to a preferred role in the hospital setting.
Zhi Lok 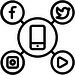	Zhi Lok was offered a room in an allied health clinic in a regional city (~2.5‐hour drive from home). They spent time creating advertising material and visiting general practice clinics to generate client referrals. During the course of the study, they did not receive a single client referral.
Steph 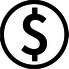	Steph applied for, and was offered, a position in a large private dietetic practice owned by a dietitian. After reading the contract and noticing the low rates of pay and high level of independence required in generating client referrals, they decided to turn the position down.

^a^ Names are pseudonyms.

Theme 1 was ‘private practice skills were lacking’. Participants reported that they felt inadequately prepared for employment in private practice, mainly due to a lack of exposure to this sector during their education. They explained that hospital experiences dominated their placements, with little translation to the private sector. Participants described anxiety around private practice work, highlighting a perceived lack of skills and confidence. They indicated that their preparedness could have been enhanced through more business skills education. Additionally, they identified a lack of skill in creating rapport and trust with clients to establish long‐term relationships.

“…I don't know how to market myself, grow a business… we had a one‐hour lecture on it. It didn't prepare us for private practice, which is where a lot of the jobs are…knowing how to [run a private practice] would be amazing and knowing how to build long‐lasting relationships where the clients come back week after week would be really good…” (Caitlin, audio‐diary)

Participants reported feeling ill‐prepared for business administration, including marketing themselves to employers and clients. In particular, they indicated a lack of confidence in invoicing clients and communicating with referrers, such as general practitioners and other allied health professionals.

“How do I write an invoice to a client?… How do I do marketing? …if I have a meeting with a GP clinic, how do I actually get them on board? How do I do tax? Like, I have no idea…” (Pat, entrance interview)

Participants identified that having a speciality area (such as eating disorders) was one way to differentiate themselves in the private market, but they also indicated unpreparedness for such speciality areas.

Theme 2 was ‘making a living was challenging’. Participants reported that contract employment was insecure, often generating inadequate income to support themselves. They highlighted that income was dependent on their ability to build a client base, which they did not feel confident achieving.

“… [I'm] just worried about whether I can get enough money and patients to support myself. I feel I'll be a lot more worried [laughs] if I did private practice…because I'm always constantly need[ing] to find money to feed myself [laughs].” (Zhi Lok, entrance interview)

They also expressed concern about financial stress caused by clients cancelling or failing to attend appointments. Participants expressed the opinion that large businesses took advantage of recent graduates with low rates of pay per client and no income while establishing a client base. However, the one participant who took on this type of role expressed gratitude for the experience it provided, leveraging this to obtain a preferred hospital position (Table 1, Pat). Within their audio‐diaries, Steph described feeling conflicted about declining a private practice position because of the low pay rates and the high level of independence required (Table 1, Steph), but missing the opportunity to obtain valuable employment experience:

“I was really shaken [when they offered me the position], because I went through this whole rollercoaster of ‘I'd be stupid not to take a dietetics job, because it would be great to get experience’. But really, the job's not what I wanted at all. I don't want to work in private practice, especially as a first job, and I may as well have just set up my own private practice and got the full money for each consult…”

Working in private practice was described as involving long hours and duties that did not directly generate income. One participant discussed unpaid work while preparing for a private practice position, including meeting with general practice clinic managers and creating advertising material to encourage client referrals, like pamphlets and social media blogs and posts. They recounted not generating a single referral during the data collection period (Table 1, Zhi Lok). Pat, who was employed in a private practice, had no access to reception staff and needed to set up and confirm their own client appointments. They described this as a hard and tedious process, requiring after‐hours work without a commensurate increase in income.

Theme 3 was ‘support was needed’. Participants indicated that professional support and clinical supervision from their employer was essential to their success in private practice, particularly if their workload included complex client conditions. One participant expressed concern that without supervision from an experienced dietitian, they may fail to develop the required skills. Participants believed that professional support would be provided in dietitian‐owned companies employing several dietitians but reportedly found the level offered to be inadequate. This was discussed by Pat during their exit interview, who resigned after several months of feeling inadequately supported (Table 1, Pat):

“…one of the main reasons [I resigned] was because I couldn't see the person [manager] face‐to‐face, because I was working two hours from the closest manager, so it was all over the phone. Whilst she would have an hour a week, I felt like I needed more [support] than that at that time.”

Participants valued social support and business advice from family, friends, university lecturers and mentors when deciding to take on private practice roles.

“I was having phone calls all day and meeting with people all day, helping me, giving me their opinion and their expertise in the area of business and dietetics and life and family…although I felt unprepared in the situation [of deciding whether to accept the position], looking back, I feel very lucky that I had people around me that could help me navigate that week.” (Steph, audio‐diary)

All 18 universities responded to the survey (100% response). Of these, 14 (78%) offered private practice placements, either within an external business (*n* = 7; 39%), a student‐led clinic (*n* = 3; 17%), an internship within an external business (*n* = 2; 11%) or ‘other’ (*n* = 2; 11%). Four universities (22%) did not offer any form of private practice placement. Placements were offered as either an elective (*n* = 8; 44%) or a core component (*n* = 6; 33%). They varied in length and could be 10 days or less (*n* = 7; 39%), 20 days (*n* = 4; 22%), or 25–40 days (*n* = 4; 22%) (one university offered two different time frames). Of the 14 universities offering private practice placements, 5 (36%) planned to change current offerings by: increasing available places; engaging a large private practice company; or using private practice as a replacement or backup for hospital placements. Those universities not currently offering private practice placements had no plans to offer them in the future (*n* = 4; 22%).

Private practice was considered by some to be unsuitable for new graduate employment and therefore not an essential placement setting for students. Universities expressed concern that private practice placements lacked the opportunity for students to demonstrate competencies due to limited client contact hours, particularly with external private practice providers. Another concern was that finding placement partners could be difficult due to a perceived lack of benefits to the business. One university reported previously trying to utilize a private practice placement, but was told that their accreditation renewal would face additional scrutiny. However, another hoped to incorporate more private practice placements in the future as recent changes to accreditation standards allowed greater flexibility.

**FIGURE 2:**
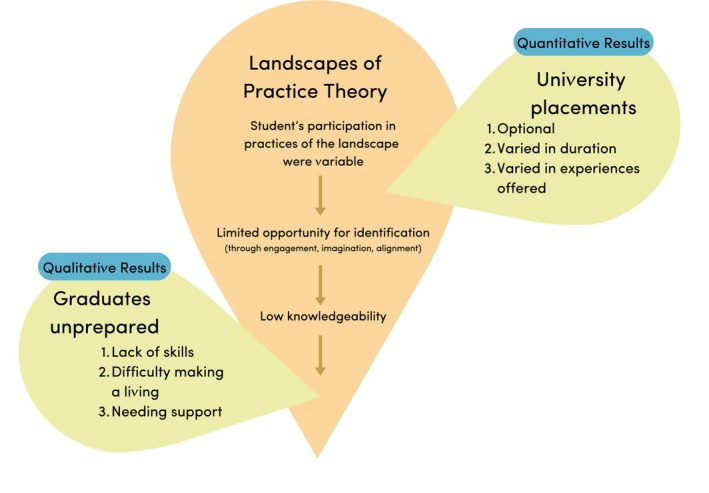
Interpretation of the study findings about private practice preparedness and placement experiences with respect to the landscapes of practice theory.

Despite concerns about limited client contact hours, some educators expressed positive views about student skill development during private practice placements, including business skills, marketing, and communication. Educators expressed that preparing students for private practice employment was important due to increasing rates of employment in the setting and the finite availability of hospital placements. Educators indicated wanting to offer students more exposure to client contact hours within this setting. Implementing a student demonstration clinic to provide placement experiences meeting the required competencies for medical nutrition therapy was described as a potential way forward. Collaborating with other university programs to learn about successfully implementing private practice placements was also mentioned. Figure 2 presents the interpretation of the qualitative and quantitative results with respect to the landscapes of practice theory discussed further below.

## 4 DISCUSSION

This study qualitatively explored dietetics graduates' experiences of private practice employment and employability and elaborated on these findings by surveying Australia and New Zealand dietetics program directors on the utilisation of private practice placements. Qualitative data found graduates were unprepared for this sector, with themes illustrating that they lacked private practice skills, found making a living challenging, and needed support. These findings were somewhat explained by the survey showing that private practice placements were typically optional, of varying durations and potentially lacking opportunities for client contact. While educators recognised the need to look to the future of incorporating private practice placements, accreditation fears, as well as anxieties about students not developing required skills were reasons for not utilising this setting.

Concerning Theme 1, graduates reported feeling unprepared for private practice, citing a lack of exposure to this setting. This has been reported briefly elsewhere,^3^, ^4^, ^5^ and may be due to inconsistent private practice experiences across universities, perhaps a legacy of previous accreditation standards not mandating this placement setting.^37^ As placements commonly occur within hospital, food service, and public health settings,^37^ dietetics students navigate multiple workplace contexts and the boundaries between them.^19^ Dietetics graduates report preparedness for entry‐level practice, yet cite a lack of private practice skills.^3^ The landscapes of practice theory suggest that while competence can translate across settings, developing an identity as a capable professional in one context can be challenging to translate into another.^19^ Without robust and consistent experiences in private practice, students' ability to develop the knowledgeability required, through identification, imagination, and alignment,^19^ for successfully navigating this employment setting may be limited.

Theme 2 demonstrated that participants found it difficult to make a living as new graduates in private practice, with several contributing factors. Graduates have previously reported that pressure from referrers to only charge the government insurance rebate with no additional expense to clients and lengthy consultations resulted in lower income.^7^ The income challenges reported by graduates in the current study are complex and multifaceted. Ways to attenuate these issues are likely part of the body of knowledge held by existing members of the landscape of practice.^19^ Those who inhabit the landscape hold information about the evolving body of knowledge and its application to different contexts through boundary crossing.^19^ Private practice placements provide the opportunity for students to engage with knowledge holders and experience knowledge application in various contexts.^19^ Spending time within the landscape and engaging with the knowledge holders would likely improve graduates' confidence in navigating income challenges. There is no ideal duration for placements, as it is the richness of experiences that influences identification with and socialisation into the landscape.^19^ Two days observing a clinician is unlikely to provide sufficient opportunity for in‐depth engagement to build knowledgeability within the landscape. Students would likely benefit from spending more time on placement in private practice than is currently provided.

In Theme 3, participants reported a lack of support being pivotal in their unpreparedness for private practice. Graduates have reported isolation when working in private practice, more so than when working in the hospital setting.^6^ Experienced practitioners also report a lack of support when starting out in private practice.^10^ An important aspect of landscapes of practice is the socialisation that occurs between newcomers and existing members.^19^ Engagement with existing members of the landscape allows students to navigate from the periphery and build ties with core members of the community, enabling multi‐membership across the landscape of practice.^19^ If students are not given the opportunity to spend time within the setting, they may not be able to develop supportive relationships and build networks to draw upon later.

The survey identified momentum for change to incorporate private practice placements, but program directors noted that this was hindered by previous accreditation standards. They also indicated that engaging businesses to host students was challenging. Experienced private practice practitioners see placements as an important way to prepare the graduate workforce and as a way for them to give back to the profession.^10^ Co‐designing placements with business owners, educators, and graduates to ensure that these placements are beneficial to all parties could be a strategy to overcome these barriers while ensuring students are able to demonstrate the required competencies.^38^

Our study's strengths include a rigorous team‐based analysis, voluminous longitudinal qualitative data, and surveying a complete sample of dietetics education providers across Australia and New Zealand. Limitations are that we only sampled dietetics graduates from one Australian university, where not all students experience authentic client interactions in private practice settings. We did not collect data on exposure to private practice placements from participants; however, dietetics graduates have previously reported a lack of private practice skills in larger scale quantitative research,^3^ suggesting this issue persists for many. While the sample for the qualitative phase was largely homogenous (i.e., female, white), it is consistent with the dietetics graduate workforce.^3^ We collected qualitative data prior to the COVID‐19 pandemic, and we acknowledge a gap in the time between qualitative and quantitative data collection. It is unclear whether and how the private practice landscape has since changed, potentially affecting the transferability of the qualitative findings. Quantitative data collection occurred as updated accreditation standards were released, and it is unclear whether and how educational practices may have since changed. Further research is required, over a longer time period, to unpack new graduates' experiences of private practice employment from other universities with more substantive private practice placements. Capturing contextual data related to private practice placements (e.g., placement duration, type of clinic, activities conducted by students, etc.) may support a more comprehensive understanding of graduates' preparedness for employment in this setting.

This study has demonstrated that private practice placements need to be a core component of dietetics education to prepare the dietetics workforce for available employment opportunities. Placements of reasonable duration, offering rich experiences that enhance graduate knowledgeability, are essential for preparing graduates for work within this landscape. Co‐designing placement experiences may address concerns regarding business models and mutual benefit to students and business owners. Building a supervision culture to adequately support graduates entering private practice is essential for ongoing socialisation into this landscape of practice. Evaluating the impact of these placements on preparedness for practice is also required.

## AUTHOR CONTRIBUTIONS

MB, CP, LJM and SG conceived and designed the study. CER, CP, LVM and EO authorised the use of the original data for secondary data analysis. EO conducted the interviews. MB, CP, LJM and SG conducted the qualitative analysis, which was cross checked by CR, EO and LVM, each of whom provided analytical insight. MB, LJM, SG and CP designed the quantitative survey and analysed the data. CER, LVM and EO cross‐checked the data analysis. MB, CP and CR drafted the manuscript and all other authors provided critical revisions.

## FUNDING INFORMATION

MB was supported by an Australian Government, Research Training Program Scholarship and Stipend.

## CONFLICT OF INTEREST STATEMENT

Lana Mitchell and Claire Palermo are Editorial Board Members of Nutrition & Dietetics. They were excluded from the peer review process and all decision‐making regarding this article. This manuscript has been managed throughout the review process by the Journal's Editor‐in‐Chief. The journal operates a blinded peer review process and the peer reviewers for this manuscript were unaware of the authors of the manuscript. This process prevents authors who also hold an editorial role to influence the editorial decisions made.

## Data Availability

The data that support the findings of this study are available from the original research team who collected the data. Restrictions apply to the availability of these data, which were used under license for this study. Data are available from the author(s) with the permission of the original research team who collected the data.
